# Evaluation of Proenkephalin A 119–159 for liberation from renal replacement therapy: an external, multicenter pilot study in critically ill patients with acute kidney injury

**DOI:** 10.1186/s13054-023-04556-w

**Published:** 2023-07-10

**Authors:** Thilo von Groote, Felix Albert, Melanie Meersch, Raphael Koch, Joachim Gerss, Birte Arlt, Mahan Sadjadi, Christian Porschen, Peter Pickkers, Alexander Zarbock, the RICH investigators

**Affiliations:** 1 Department of Anaesthesiology, Intensive Care and Pain Medicine, University Hospital Münster, Albert-Schweitzer-Campus 1, 48149 Münster, Germany; 2 Institute of Biostatistics and Clinical Research, University of Münster, Münster, Germany; 3 SphingoTec GmbH, Hennigsdorf, Germany; 4 Department of Intensive Care Medicine, Radboud University Medical Center, Nijmegen, The Netherlands

**Keywords:** Acute kidney injury, Biomarker, Critical illness, Proenkephalin, Renal replacement therapy

## Abstract

**Introduction:**

Recent evidence suggests an association of plasma Proenkephalin A 119–159 (penKid) with early and successful liberation from continuous renal replacement therapy (CRRT) in critically ill patients with acute kidney injury. However, these exploratory results are derived from a monocentric trial and therefore require external validation in a multicenter cohort.

**Methods:**

Data and plasma samples from the “Effect of Regional Citrate Anticoagulation versus Systemic Heparin Anticoagulation During Continuous Kidney Replacement Therapy on Dialysis Filter Life Span and Mortality Among Critically Ill Patients With Acute Kidney Injury—A Randomized Clinical Trial” (RICH Trial) were used for this validation study. PenKid was measured in all plasma samples available at CRRT initiation and at day 3 of CRRT. Patients were categorized into low and high penKid groups with a cutoff at 100 pmol/l. Competing-risk time-to-event analyses were performed. Competing risk endpoints were successful and unsuccessful liberation from CRRT, the latter meaning death or initiation of a new RRT within one week of discontinuation of primary CRRT. Then penKid was compared to urinary output.

**Results:**

Low pre-CRRT penKid levels at CRRT initiation were not associated with early and successful liberation from CRRT compared to patients with high pre-CRRT penKid levels [subdistribution hazard ratio (sHR) 1.01, 95% CI 0.73–1.40, *p *= 0.945]. However, the landmark analysis on day 3 of ongoing CRRT demonstrated an association between low penKid levels and successful liberation from CRRT (sHR 2.35, 95% CI 1.45–3.81, *p *< 0.001) and an association between high penKid levels and unsuccessful liberation (sHR 0.46, 95% CI 0.26–0.80, *p *= 0.007). High daily urinary output (> 436 ml/d) was even stronger associated with successful liberation (sHR 2.91, 95% CI 1.80–4.73, *p *< 0.001) compared to penKid.

**Discussion:**

This study suggests that penKid may be a competent biomarker to monitor the recovery of kidney function during CRRT. This is in line with previous findings and investigated this concept in a multicenter cohort. Again, low penKid was associated with early and successful CRRT liberation, but was outperformed by high daily urinary output. The findings of this study now warrant further evaluation in prospective studies or a randomized controlled trial.

*Trial registration* The RICH Trial was registered at clinicaltrials.gov: NCT02669589. Registered 01 February 2016.

**Supplementary Information:**

The online version contains supplementary material available at 10.1186/s13054-023-04556-w.

## Background

When subscribing continuous renal replacement therapy (CRRT) to critically ill patients with acute kidney injury (AKI), intensivists lack suitable tools to assess kidney recovery and to determine optimal timing of therapy cessation. Unfortunately, CRRT modifies standard markers of kidney function, such as serum creatinine. While urine output might be a predictor for CRRT liberation, it has a low sensitivity, and heterogeneity of investigated thresholds, study outcomes and lack of validation studies hampers generalizability of study results [1, 2].

Recently, we investigated whether penKid may be a competent marker to assess kidney function during CRRT and found an association between low penKid values, indicating better kidney function, with early and successful liberation from CRRT. Our hypothesis was based on data that demonstrated penKid as a marker of kidney function in unstable settings, such as sepsis or critical illness [3–5]. However, our findings were only of exploratory nature and derived from a monocentric study. Hence, external and multicenter investigation of this concept was required.

## Materials and methods

This pilot study is a post-hoc analysis of the multicentric “Effect of Regional Citrate Anticoagulation versus Systemic Heparin Anticoagulation During Continuous Kidney Replacement Therapy on Dialysis Filter Life Span and Mortality Among Critically Ill Patients With Acute Kidney Injury—A Randomized Clinical Trial" (RICH Trial) [6]. The RICH Trial compared different anticoagulation strategies in critically ill patients with AKI requiring CRRT and enrolled 596 patients. Laboratory samples were stored at different time points as part of an add-on study. AKI was defined using the KDIGO 2012 definition [7]. Adult patients were included if they developed stage 3 AKI and/or an absolute CRRT indication and had at least one pre-defined condition of critical illness (severe sepsis, septic shock, shock requiring vasopressors, fluid overload).

PenKid was measured in EDTA plasma samples using the immunoluminometric sphingotest^®^ penKid^®^ assay (SphingoTec GmbH, Germany) as described previously [3]. We distinguished two competing events: “successful liberation from CRRT” and “unsuccessful liberation from CRRT”. Patients who survived and did not receive any form of RRT for at least 7 days (relapse-free period) after CRRT discontinuation were classified as successfully liberated. Patients who were not followed up for at least 7 days after discontinuation of CRRT were censored at the end of CRRT. Patients who required CRRT after day 28 were censored at day 28.

**Fig. 1:**
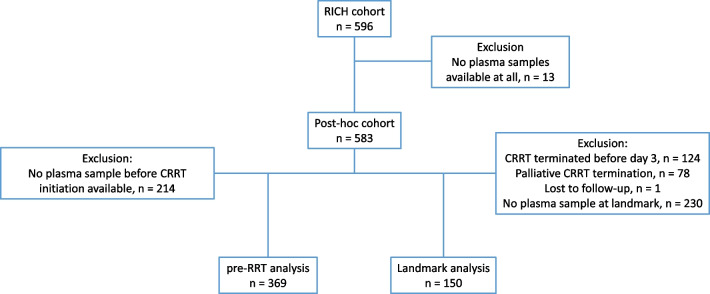
Study cohort flowchart

Figure 1 summarizes the study workflow. Sensitivity analyses were performed to test the robustness of the study findings with respect to changes in the cutoff value (Additional file 1: Fig. S1). Patients were grouped based on their penKid value before CRRT initiation (baseline) and at day 3 of ongoing CRRT (landmark), daily urinary output (below or above 436 ml/d [8]), and randomization group. We provide a detailed description of statistical methods in the Additional file 1.

## Results

### Baseline characteristics

Patient characteristics differed only with regard to serum creatinine and eGFR values at both time points (Additional file 1: Tables S1, S2). Additionally, at landmark, patients differed in age and rates of diabetes mellitus. The randomization groups were balanced in all penKid groups. (Additional file 1: Table S3).

### Establishment of the cut-offs

We hypothesized that penKid would identify patients with recovering kidney function while receiving CRRT, therefore, all cutoffs were based on the landmark time point. Sensitivity analysis of penKid demonstrated best performance and robustness for cutoffs between 90 and 105 pmol/l (Additional file 1: Fig. S1). Main analyses were repeated using the standard cutoff (89 pmol/l) (Additional file 1: Fig. S2).

The association between urinary output and successful liberation was constantly high, but strongly dependent on the cutoff (Additional file 1: Fig. S3). Therefore, an established cutoff (436 ml/d) with good performance was used.

### CRRT duration pre-CRRT analysis

**Fig. 2:**
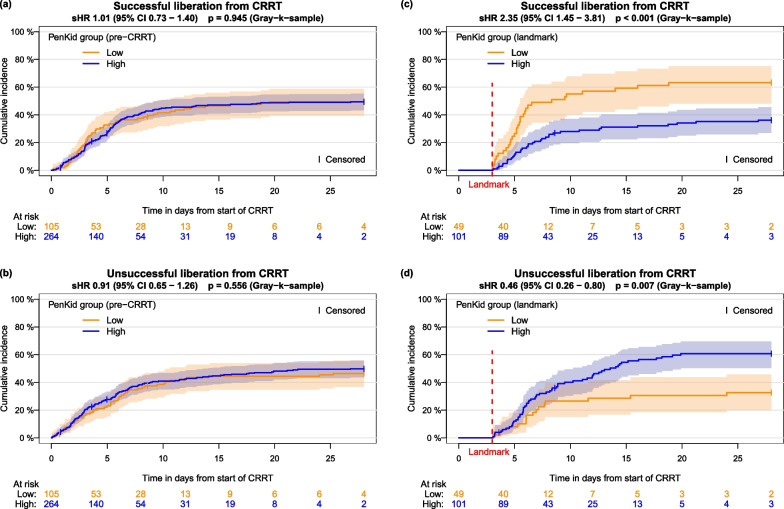
Estimated cumulative incidence functions with log–log transformed pointwise 95% confidence intervals of successful liberation from CRRT **(a)**, **(c)** and unsuccessful liberation from CRRT **(b)**, **(d)** for the groups based on penKid value (≤ 100 pmol/l, > 100 pmol/l) before CRRT and based on the value at day 3 (landmark)

In the first 28 days after initiation of CRRT, therapy was successfully discontinued in 180/369 patients (28d-cumulative incidence function (CIF) 49.4%). PenKid group was not associated with either of the two competing outcomes (Fig. 2a, b).

High urinary output on day 0 (> 436 ml/d) was associated with successful liberation from CRRT (sHR 3.02, 95% CI 2.21–4.12, *p *< 0.001), as well as a reduced rate of unsuccessful CRRT liberation (sHR 0.35, 95% CI 0.23–0.53, *p *< 0.001) (Additional file 1: Fig. S4a, b), while randomization group showed no effect on either of the competing outcomes (Additional file 1: Fig. S5a, b).

### Landmark analysis

Successful liberation from CRRT occurred considerably more often in the low penKid group (31/49, 28d-CIF 63.3% versus 36/101, 28d-CIF 36.2%, *p *= 0.003). The univariate model yields a sHR of 2.35 (95% CI 1.45–3.81, *p *< 0.001) (Fig. 2c). Unsuccessful liberations were less frequent in the low penKid group (sHR 0.46, 95% CI 0.26–0.80, *p *= 0.007) (Fig. 2d). The association between penKid and successful liberation from CRRT remained similar when adjusting for age and diabetes mellitus (sHR 2.22, 95% CI 1.36–3.62, *p *= 0.002) (Additional file 1: Table S4).

Results for urinary output, and randomization group were comparable to the pre-CRRT analysis (Additional file 1: Figs. S4c, d, S5c, d). Urinary output was even stronger associated with both competing outcomes than penKid.

### Sensitivity to changes in the relapse-free period

No substantial differences in findings were observed for different relapse-free periods, such as 2 or 90 days, although the overall proportion of successful/unsuccessful liberations from CRRT varied (Additional file 1: Figures S6–S9).

## Discussion

This is a post hoc analysis of a multicenter randomized trial investigating recent evidence of an association between low penKid values with early and successful CRRT liberation. This association was reproducible in this external cohort, however with limitations. First, the effect was only present during CRRT, but not at baseline. We hypothesize that the initiation time point influences the predictive value of the biomarker. In the RICH trial, CRRT was initiated in patients with more severe AKI (KDIGO stage 3 or absolute indication), compared to the ELAIN trial (KDIGO stage 2). This is also reflected in the higher median penKid in the RICH cohort (134.18 pmol/l), compared to the ELAIN cohort (106.95 pmol/l) at baseline.

Second, a slightly higher cutoff value to distinguish groups was more appropriate. This might be due to differences in patient populations: The RICH study investigated a mixed patient population, including surgical and non-surgical patients, while the ELAIN trial only included surgical patients Higher penKid levels in non-surgical patients are also in line with previous studies [9, 10]. Finally, the sensitivity analysis performed demonstrates a stable result with any given cutoff around 100 pmol/l.

Our study has several limitations, such as the retrospective study design and the relatively low rate of samples available at landmark analysis. Furthermore, the study protocol did not standardize termination of CRRT. Our results suggest that penKid may identify patients with higher chances of successful liberation from CRRT, however appropriate urinary output (> 436 ml/d) outperformed penKid. Data did not suggest a relevant imbalance between randomization groups (heparin or citrate anticoagulation) and penKid group.

Overall, this study emphasizes the potential of penKid to identify patients for CRRT liberation as it may assess kidney function even during CRRT. Moving forward, a prospective study is required.

## Supplementary Information

**Additional file 1:** Supplementary material.

## Data Availability

Statistical results are available from the authors upon reasonable request.

## Abbreviations

AKI: Acute kidney injury
CI: Confidence interval
CRRT: Continuous renal replacement therapy
GFR: Glomerular filtration rate
ICU: Intensive care unit
OR: Odds ratio
penKid: Proenkephalin A 119–159
RICH: Effect of regional citrate anticoagulation versus systemic heparin anticoagulation during continuous kidney replacement therapy on dialysis filter life span and mortality among critically Ill patients with acute kidney injury—a randomized clinical trial
sHR: Subdistributional hazard ratio
28d-CIF: Estimated cumulative incidence function at day 28
