# Supplementary material for: Evaluation of Proenkephalin A 119–159 for liberation from renal replacement therapy: an external, multicenter pilot study in critically ill patients with acute kidney injury
Source: Crit Care. 2023 Jul 10;27:276. doi: 10.1186/s13054-023-04556-w (PMC10332055; doi:10.1186/s13054-023-04556-w)
Supplement: Supplementary file 1 — Additional file 1: Supplementary material. [file 13054_2023_4556_MOESM1_ESM.pdf]

Evaluation of Proenkephalin A 119-159 for liberation from renal replacement therapy – an external, multicenter pilot study in critically ill patients with acute kidney injury

# - Supplementary material -

Contents

Figure S1: Sensitivity analysis of penKid cut-off 3

Figure S2: Cumulative incidences separated by penKid group at baseline (pre-CRRT) and landmark time point (cut-off 89 pmol/l, relapse-free period 7 days) 4

Figure S3: Sensitivity analysis of daily urinary output cut-off 5

Figure S4: Cumulative incidences separated by urinary output group at baseline (pre-CRRT) and landmark time point (cut-off 436 ml/d, relapse-free period 7 days) 6

Figure S5: Cumulative incidences separated by randomization group (heparin versus citrate anticoagulation) at baseline (pre-CRRT) and landmark time point (relapse-free period 7 days) 7

Figure S6: Cumulative incidences separated by penKid group at baseline (pre-CRRT) and landmark time point (cut-off 100 pmol/l, relapse-free period 2 days) 8

Figure S7: Cumulative incidences separated by penKid group at baseline (pre-CRRT) and landmark time point (cut-off 100 pmol/l, relapse-free period 90 days) 9

Figure S8: Cumulative incidences separated by urinary output group at baseline (pre-CRRT) and landmark time point (cut-off 436 ml/d, relapse-free period 2 days) 10

Figure S9: Cumulative incidences separated by urinary output group at baseline (pre-CRRT) and landmark time point (cut-off 436 ml/d, relapse-free period 90 days) 11

Table S1: Patient characteristics (enrollment time point) **12**

Table S2: Patient characteristics landmark analysis (day 3 of CRRT) **13**

Table S3: Cross-table of penKid groups and randomization groups with column percentages **14**

Table S4: Multivariable Fine & Gray regression predicting "successful liberation from CRRT" at landmark analysis **14**

Statistical analysis **15**

Figure S1: Sensitivity analysis of penKid cut-off

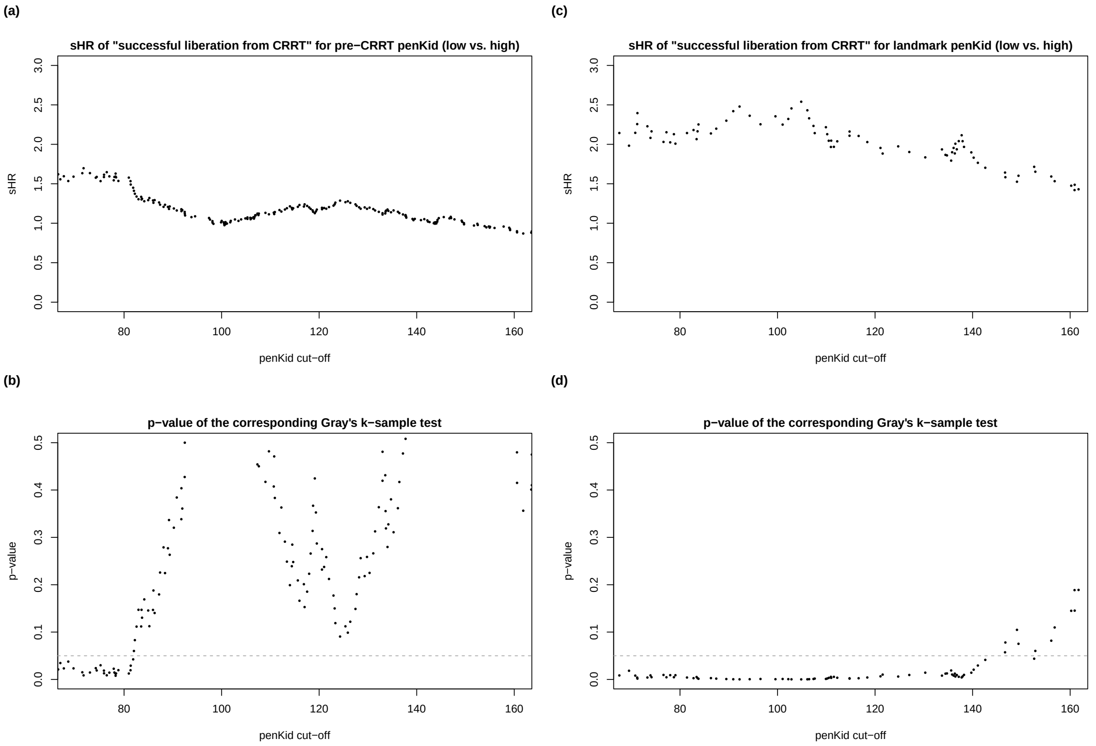

Figure S1: Estimated sHR for "successful liberation from CRRT" between **(a)** pre-CRRT and **(c)** landmark penKid groups (low vs. high) as a function of all possible penKid cut-off values between 70 - 160 pmol/l present in the data. **(b)**, **(d)** p-value of the corresponding Gray's k-sample test (H_0 : sHR = 1).

Figure S2: Cumulative incidences separated by penKid group at baseline (pre-CRRT) and landmark time point (cut-off 89 pmol/l, relapse-free period 7 days)

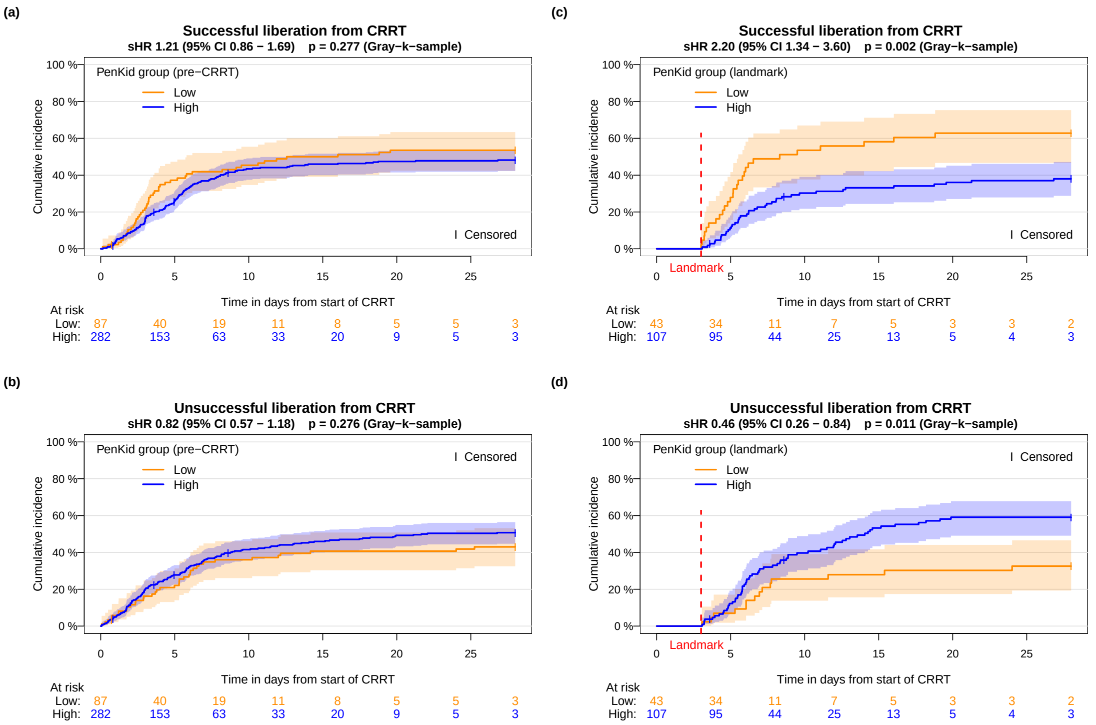

Figure S2: Estimated cumulative incidence functions with log-log transformed pointwise 95% confidence intervals of **(a)**, **(c)** successful liberation from CRRT and **(b)**, **(d)** unsuccessful liberation from CRRT for the groups based on penKid value ($\leq$ 89 pmol/l vs. $>$ 89 pmol/l) before CRRT and based on the value at day 3 (landmark). The required relapse-free period after CRRT liberation to be classified as successfully liberated was set to 7 days.

Figure S3: Sensitivity analysis of daily urinary output cut-off

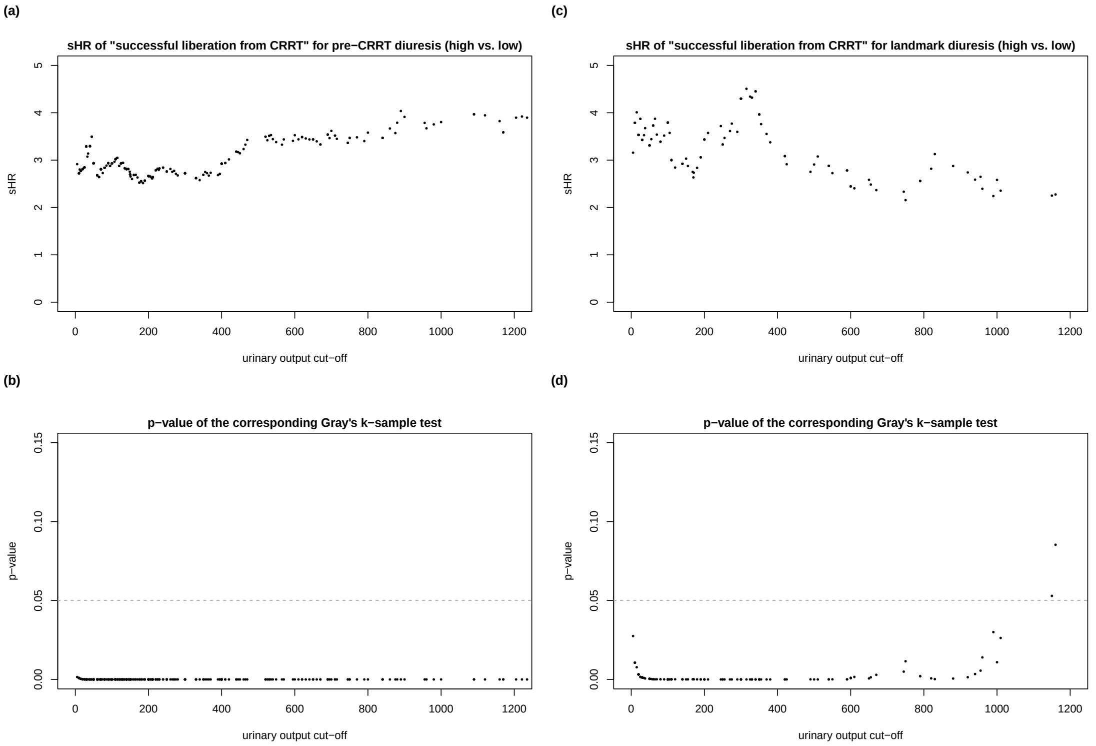

Figure S3: Estimated sHR for "successful liberation from CRRT" between **(a)** pre-CRRT and **(c)** landmark diuresis groups (high vs. low) as a function of all possible urinary output cut-off values between 0 - 1200 ml/d present in the data. **(b), (d)** p-value of the corresponding Gray's k-sample test (H_0 : sHR $= 1$).

Figure S4: Cumulative incidences separated by urinary output group at baseline (pre-CRRT) and landmark time point (cut-off 436 ml/d, relapse-free period 7 days)

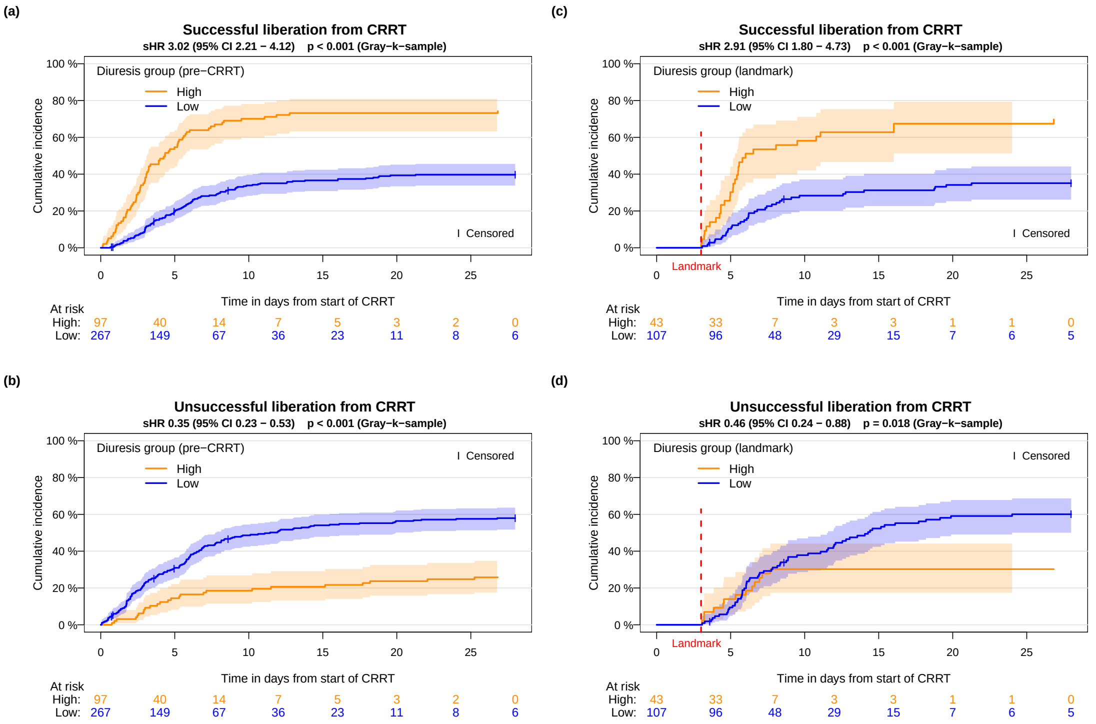

Figure S4: Estimated cumulative incidence functions with log-log transformed pointwise 95% confidence intervals of **(a)**, **(c)** successful liberation from CRRT and **(b)**, **(d)** unsuccessful liberation from CRRT for the groups based on daily urinary output (> 436 ml/d vs. ≤ 436 ml/d) before CRRT and based on the value at day 3 (landmark). Urinary output on day 0 was recorded in 364/369 patients. Urinary output on day 3 was measured in all 150 landmark patients. The required relapse-free period after CRRT liberation to be classified as successfully liberated was set to 7 days.

Figure S5: Cumulative incidences separated by randomization group (heparin versus citrate anticoagulation) at baseline (pre-CRRT) and landmark time point (relapse-free period 7 days)

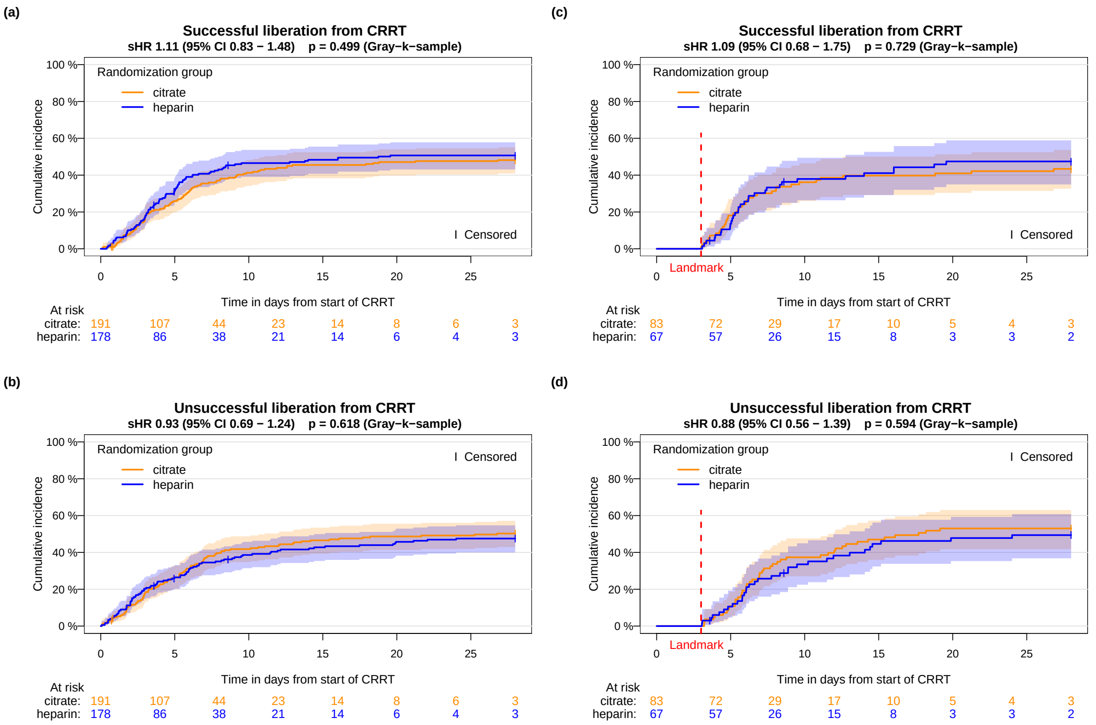

Figure S5: Estimated cumulative incidence functions with log-log transformed pointwise 95% confidence intervals of **(a)**, **(c)** successful liberation from CRRT and **(b)**, **(d)** unsuccessful liberation from CRRT for the randomization groups of the RICH trial at baseline (pre-CRRT) and landmark time point. The required relapse-free period after CRRT liberation to be classified as successfully liberated was set to 7 days.

Figure S6: Cumulative incidences separated by penKid group at baseline (pre-CRRT) and landmark time point (cut-off 100 pmol/l, relapse-free period 2 days)

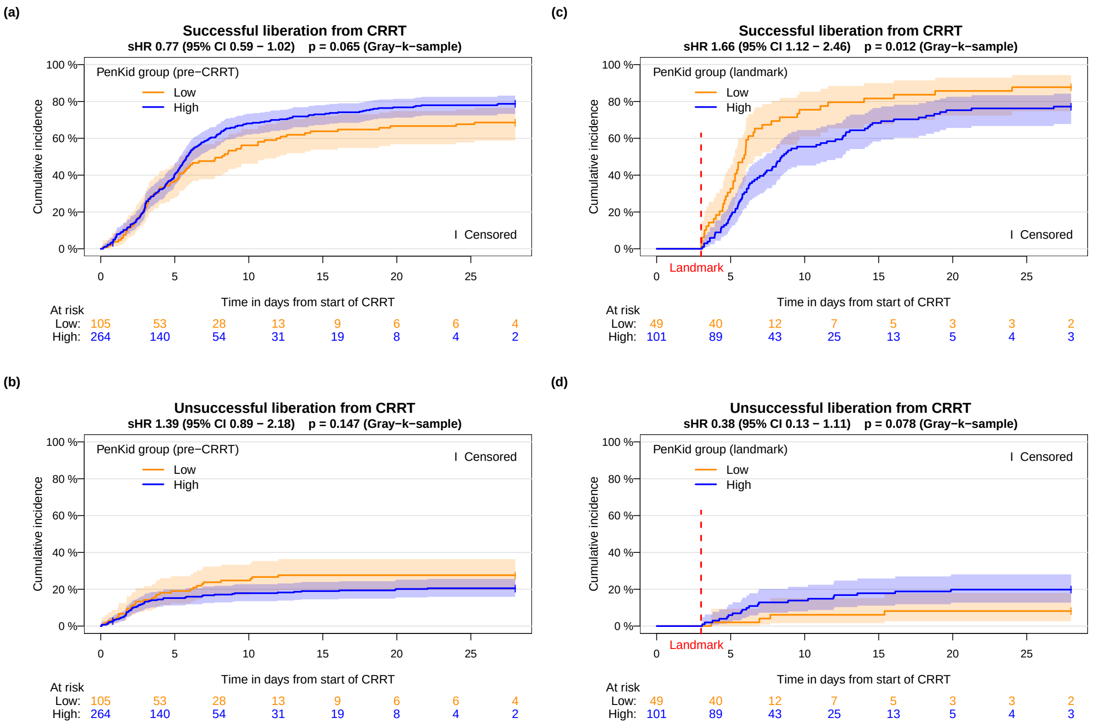

Figure S6: Estimated cumulative incidence functions with log-log transformed pointwise 95% confidence intervals of **(a)**, **(c)** successful liberation from CRRT and **(b)**, **(d)** unsuccessful liberation from CRRT for the groups based on penKid value ($\leq$ 100 pmol/l vs. $>$ 100 pmol/l) before CRRT and based on the value at day 3 (landmark). The required relapse-free period after CRRT liberation to be classified as successfully liberated was set to 2 days.

Figure S7: Cumulative incidences separated by penKid group at baseline (pre-CRRT) and landmark time point (cut-off 100 pmol/l, relapse-free period 90 days)

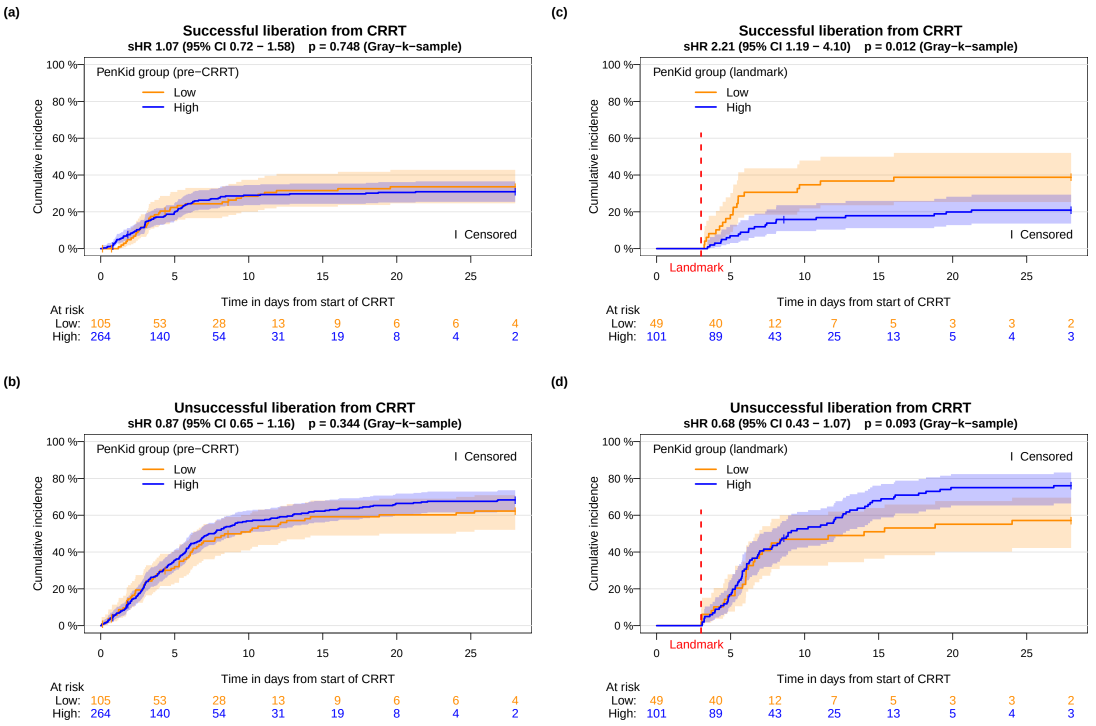

Figure S7: Estimated cumulative incidence functions with log-log transformed pointwise 95% confidence intervals of **(a)**, **(c)** successful liberation from CRRT and **(b)**, **(d)** unsuccessful liberation from CRRT for the groups based on penKid value ($\leq$ 100 pmol/l vs. $>$ 100 pmol/l) before CRRT and based on the value at day 3 (landmark). The required relapse-free period after CRRT liberation to be classified as successfully liberated was set to 90 days.

Figure S8: Cumulative incidences separated by urinary output group at baseline (pre-CRRT) and landmark time point (cut-off 436 ml/d, relapse-free period 2 days)

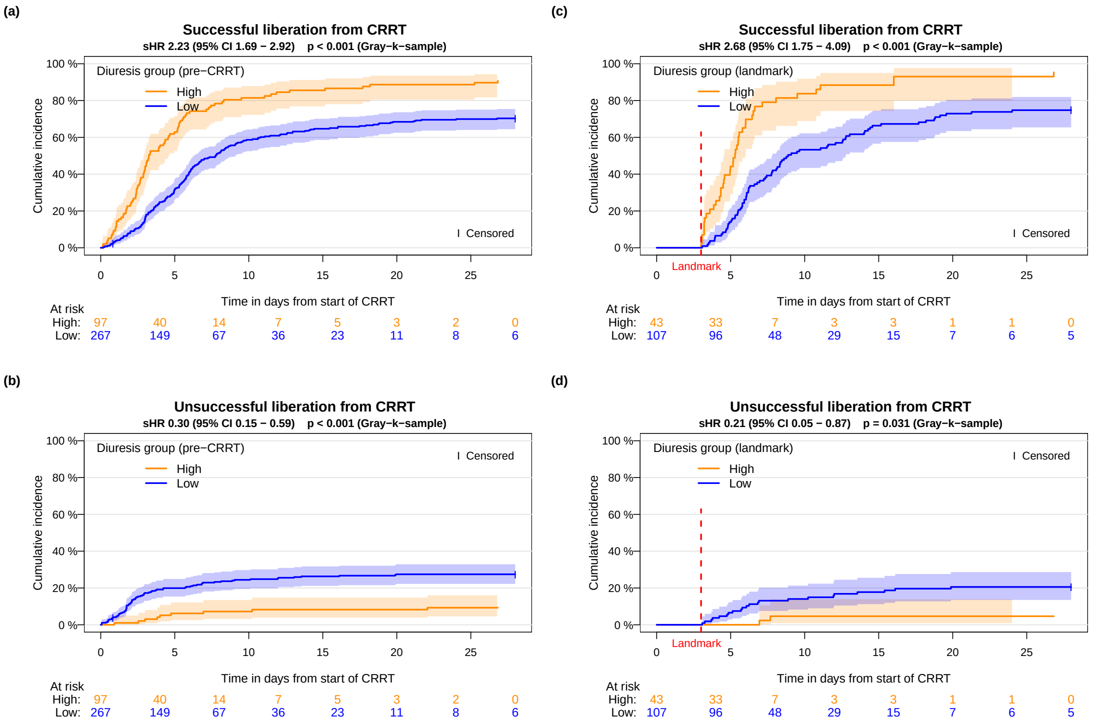

Figure S8: Estimated cumulative incidence functions with log-log transformed pointwise 95% confidence intervals of **(a)**, **(c)** successful liberation from CRRT and **(b)**, **(d)** unsuccessful liberation from CRRT for the groups based on daily urinary output ($>$ 436 ml/d vs. $\leq$ 436 ml/d) before CRRT and based on the value at day 3 (landmark). Urinary output on day 0 was recorded in 364/369 patients. Urinary output on day 3 was measured in all 150 landmark patients. The required relapse-free period after CRRT liberation to be classified as successfully liberated was set to 2 days.

Figure S9: Cumulative incidences separated by urinary output group at baseline (pre-CRRT) and landmark time point (cut-off 436 ml/d, relapse-free period 90 days)

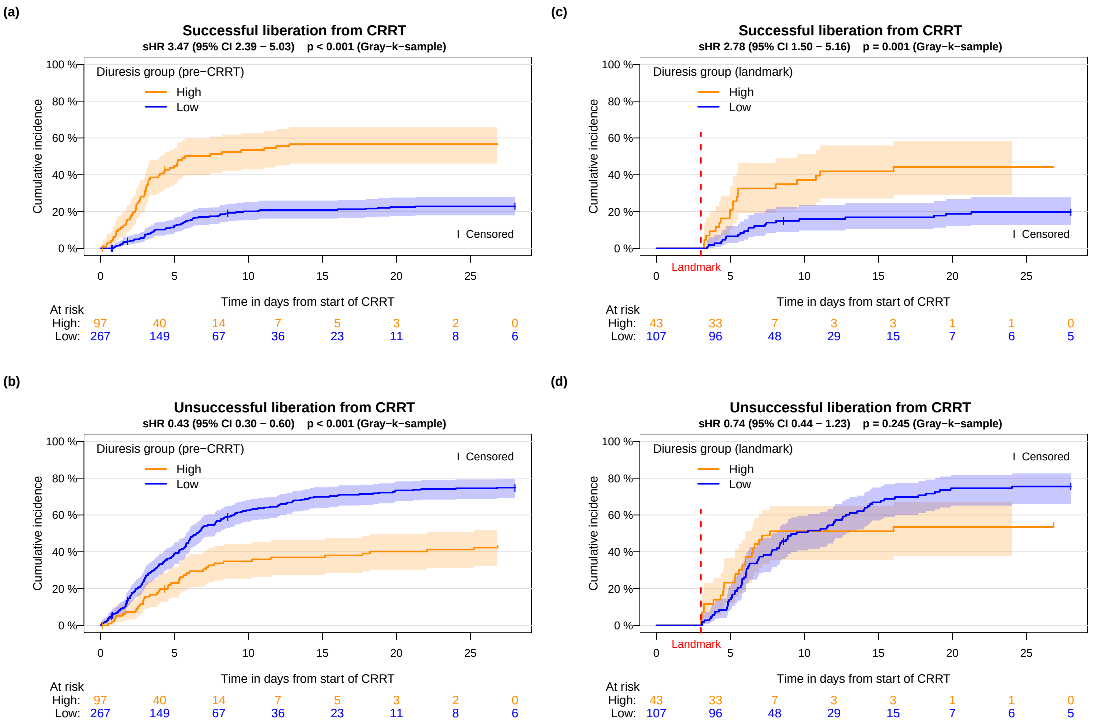

Figure S9: Estimated cumulative incidence functions with log-log transformed pointwise 95% confidence intervals of **(a)**, **(c)** successful liberation from CRRT and **(b)**, **(d)** unsuccessful liberation from CRRT for the groups based on daily urinary output ($>$ 436 ml/d vs. $\leq$ 436 ml/d) before CRRT and based on the value at day 3 (landmark). Urinary output on day 0 was recorded in 364/369 patients. Urinary output on day 3 was measured in all 150 landmark patients. The required relapse-free period after CRRT liberation to be classified as successfully liberated was set to 90 days.

Table S1: Patient characteristics (enrollment time point)

Variable	Total (n=369)	Low penKid (≤100 pmol/l) (n=105)	High penKid (>100 pmol/l) (n=264)	p-value
**Sex**				
Male	250 (67.8%)	74 (70.5%)	176 (66.7%)	0.538[a]
Female	119 (32.2%)	31 (29.5%)	88 (33.3%)	
**Age (years)**				
Median (Q1, Q3)	69.0 (61.0, 77.0)	68.0 (59.0, 76.0)	70.0 (62.0, 78.0)	0.067[b]
Mean (SD)	67.9 (12.1)	66.4 (11.7)	68.5 (12.2)	
**Creatinine (mg/dl)**				
Median (Q1, Q3)	2.30 (1.65, 3.00)	1.80 (1.38, 2.53)	2.40 (1.90, 3.13)	<0.001[b]
Mean (SD)	2.47 (1.14)	2.09 (1.07)	2.61 (1.13)	
Missing	20 (5.4%)	9 (8.6%)	11 (4.2%)	
**Estimated GFR (ml/min/1.73m^2)**				
Median (Q1, Q3)	26.0 (19.3, 36.7)	34.9 (24.4, 49.8)	23.8 (18.0, 33.1)	<0.001[b]
Mean (SD)	30.1 (15.9)	38.4 (19.6)	26.9 (13.1)	
Missing	20 (5.4%)	9 (8.6%)	11 (4.2%)	
**SOFA score**				
Median (Q1, Q3)	11.0 (9.00, 13.0)	11.0 (10.0, 13.0)	11.0 (9.00, 13.0)	0.248[b]
Mean (SD)	11.1 (2.93)	11.3 (2.78)	11.0 (2.99)	
Missing	24 (6.5%)	5 (4.8%)	19 (7.2%)	
**APACHE II**				
Median (Q1, Q3)	27.0 (23.0, 32.0)	27.0 (23.0, 31.8)	28.0 (23.0, 32.0)	0.404[b]
Mean (SD)	28.0 (6.92)	27.3 (6.74)	28.2 (6.99)	
Missing	42 (11.4%)	11 (10.5%)	31 (11.7%)	
**Hypertension**				
No	108 (29.3%)	34 (32.4%)	74 (28.0%)	0.446[a]
Yes	259 (70.2%)	70 (66.7%)	189 (71.6%)	
Missing	2 (0.5%)	1 (1.0%)	1 (0.4%)	
**Diabetes**				
No	261 (70.7%)	74 (70.5%)	187 (70.8%)	1.000[a]
Yes	108 (29.3%)	31 (29.5%)	77 (29.2%)	
**Chronic obstructive pulmonary disease (COPD)**				
No	311 (84.3%)	91 (86.7%)	220 (83.3%)	0.526[a]
Yes	58 (15.7%)	14 (13.3%)	44 (16.7%)	
**Vasopressors**				
No	34 (9.2%)	5 (4.8%)	29 (11.0%)	0.073[a]
Yes	334 (90.5%)	100 (95.2%)	234 (88.6%)	
Missing	1 (0.3%)	0 (0%)	1 (0.4%)	

Table S1: Patients characteristics at enrollment; all variables were collected at the time of randomization except for estimated GFR and creatinine, which were measured at the start time of CRRT.
[a]Fisher's exact test comparing the low and high pre-CRRT penKid group. [b]Mann–Whitney U test comparing the low and high pre-CRRT penKid group. Abbreviations: APACHE II, Acute Physiology and Chronic Health Evaluation II, GFR, Glomerular Filtration Rate; SD, Standard Deviation; SOFA, Sequential Organ Failure Assessment

Table S2: Patient characteristics landmark analysis (day 3 of CRRT)

Variable	Total (n=150)	Low penKid (≤100 pmol/l) (n=49)	High penKid (>100 pmol/l) (n=101)	p-value
**Sex**				
Male	102 (68.0%)	33 (67.3%)	69 (68.3%)	1.000[a]
Female	48 (32.0%)	16 (32.7%)	32 (31.7%)	
**Age (years)**				
Median (Q1, Q3)	69.0 (61.0, 76.0)	64.0 (54.0, 72.0)	70.0 (63.0, 78.0)	<0.001[b]
Mean (SD)	66.8 (13.3)	61.6 (14.5)	69.3 (12.0)	
**Creatinine (mg/dl)**				
Median (Q1, Q3)	1.40 (1.00, 2.10)	1.10 (0.900, 1.60)	1.50 (1.10, 2.20)	0.001[b]
Mean (SD)	1.60 (0.806)	1.32 (0.711)	1.74 (0.817)	
Missing	1 (0.7%)	0 (0%)	1 (1.0%)	
**Estimated GFR (ml/min/1.73m^2)**				
Median (Q1, Q3)	45.5 (30.7, 69.1)	58.1 (41.9, 86.1)	39.0 (28.1, 55.4)	<0.001[b]
Mean (SD)	51.4 (26.4)	65.0 (30.8)	44.8 (21.2)	
Missing	1 (0.7%)	0 (0%)	1 (1.0%)	
**SOFA score**				
Median (Q1, Q3)	11.0 (9.00, 13.0)	10.0 (9.00, 13.0)	11.0 (9.00, 13.0)	0.550[b]
Mean (SD)	11.2 (2.90)	11.1 (3.07)	11.2 (2.83)	
Missing	10 (6.7%)	4 (8.2%)	6 (5.9%)	
**APACHE II**				
Median (Q1, Q3)	26.0 (23.0, 31.5)	25.0 (23.0, 28.8)	26.0 (23.0, 32.0)	0.161[b]
Mean (SD)	27.3 (7.07)	25.7 (5.86)	28.1 (7.51)	
Missing	23 (15.3%)	7 (14.3%)	16 (15.8%)	
**Hypertension**				
No	48 (32.0%)	19 (38.8%)	29 (28.7%)	0.195[a]
Yes	101 (67.3%)	29 (59.2%)	72 (71.3%)	
Missing	1 (0.7%)	1 (2.0%)	0 (0%)	
**Diabetes**				
No	113 (75.3%)	43 (87.8%)	70 (69.3%)	0.015[a]
Yes	37 (24.7%)	6 (12.2%)	31 (30.7%)	
**Chronic obstructive pulmonary disease (COPD)**				
No	133 (88.7%)	47 (95.9%)	86 (85.1%)	0.058[a]
Yes	17 (11.3%)	2 (4.1%)	15 (14.9%)	
**Vasopressors**				
No	16 (10.7%)	3 (6.1%)	13 (12.9%)	0.267[a]
Yes	134 (89.3%)	46 (93.9%)	88 (87.1%)	

Table S2: Patient characteristics of landmark patients, i.e. patients still receiving CRRT at day 3. All variables were collected at the time of randomization except for estimated GFR and creatinine, which were measured at day 3 of CRRT. [a]Fisher's exact test comparing the low and high landmark penKid group. [b]Mann–Whitney U test comparing the low and high landmark penKid group. Abbreviations: APACHE II, Acute Physiology and Chronic Health Evaluation II, GFR, Glomerular Filtration Rate; SD, Standard Deviation; SOFA, Sequential Organ FailureAssessment

Table S3: Cross-table of penKid groups and randomization groups with column percentages

	Pre-CRRT		Landmark	
	Low penKid	High penKid	Low penKid	High penKid
Citrate anticoagulation	50 (0.48)	141 (0.53)	30 (0.61)	53 (0.52)
Heparin anticoagulation	55 (0.52)	123 (0.47)	19 (0.39)	48 (0.48)

Table S4: Multivariable Fine & Gray regression predicting "successful liberation from CRRT" at landmark analysis

**Variable**	**sHR (95% CI)**	***p***
Landmark penKid group (low vs. high)	2.22 (1.36, 3.62)	0.002
Age (in 10 years)	0.91 (0.76, 1.08)	0.27
Diabetes mellitus (yes vs. no)	1.05 (0.58, 1.88)	0.88

p-values are from Gray's k-sample test.

Statistical analysis

Statistical analyses were conducted using R (Version R-4.1.2 for Windows)[i] and the publicly available packages *ComparisonCR, dplyr, haven, pROC, prodlim, rstatix, survival, survminer,* and *table1* were used. All analyses were conducted as exploratory analyses of hypothesis generation and were therefore not adjusted for multiple testing. All p-values and confidence limits were two-sided and were intended to be exploratory, not confirmatory. In this exploratory sense, p-values ≤ 0.05 were considered as statistically significant.

Baseline variables were assessed and as applicable, frequencies, percentages, medians, quartiles, means, standard deviations, and p-values were calculated. To compare baseline characteristics between pre-CRRT penKid groups (low vs. high), Fisher's exact test was used to compare categorical variables. Continuous variables were compared using the Mann-Whitney U test. Group characteristics were evaluated in the same way at the time of landmark analysis (day 3 of CRRT).

To answer our research question, we investigated the association of the pre-CRRT penKid group (penKid $\leq$ 100 pmol/l vs. penKid $>$ 100 pmol/l) with the time to liberation from CRRT considering two competing events: successful and unsuccessful liberation from CRRT. Patients who survived and did not receive any form of RRT for at least 7 days (relapse-free period) after CRRT discontinuation were classified as successfully liberated. Patients who died within 7 days after CRRT discontinuation or who started a new RRT during this period were classified as unsuccessfully liberated. Patients who were not followed up for at least 7 days after discontinuation of CRRT were censored at the end of CRRT. Patients who required CRRT after day 28 were censored at day 28.

For both competing outcomes, we estimated the cumulative incidence (CIF) using the Aalen-Johansen estimator[ii]. Gray's k-sample test was applied to compare the cumulative incidence of the corresponding event type between penKid groups[iii]. Comparisons of the cumulative incidence functions at fixed time points were performed with the methods proposed by Chen at al. using Gaynor's variance and log-log transformed cumulative incidence functions[iv]. To quantify the association between the penKid group and the incidence of each competing event, we fitted two univariate Fine and Gray models, resulting in an estimate of the subdistribution hazard ratio (sHR) for each competing event[v].

To investigate the predictive power of penKid also in patients during ongoing CRRT, we performed a landmark analysis on day 3 of CRRT. For this purpose, we selected all

---

[i] R Core Team (2022) R: A language and environment for statistical computing, R Foundation for Statistical Computing, Vienna, Austria, URL: http://R-project.org/. 2021.

[ii] Aalen O, Johansen S (1978). An empirical transition matrix for non-homogeneous Markov chains based on censored observations. *Scand J Stat. 5:* 141–50.

[iii] Gray R. (1988). A class of k-sample tests for comparing the cumulative incidence of a competing risk. *Ann Stat*; 16:1141–54.

[iv] Chen J, Hou Y, Chen Z. Statistical inference methods for cumulative incidence function curves at a fixed point in time. *Commun Stat Simul Comput.* 2018; 49: 79- 94.

[v] Fine JP, Gray RJ (1999). A proportional hazards model for the subdistribution of a competing risk. *Journal of the American Statistical Association*; 94:496–509.

patients who were still at risk (i.e. receiving CRRT) on day 3 of CRRT and defined their penKid measurement on day 3 as the landmark penKid value. We then repeated the statistical analyses described above with this landmark penKid value for the time interval from day 3 to day 28, again dividing the patients into two groups with low and high landmark penKid values, using the same cut-off value of 100 pmol/l as before. Event times were thus recalculated starting from the landmark time point. In order to compare both analyses (pre-CRRT and landmark) visually, the same time axis was used in the figures.

The choice of the penKid cut-off value was partially justified by a sensitivity analysis of the landmark analysis. To do so, we fitted Fine & Gray models for "successful liberation from CRRT" of the above type for each possible and clinically meaningful penKid cut-off value present in the data and plotted the associated sHR and *p*-value against the cut-off. We repeated the sensitivity analysis for the pre-CRRT penKid analysis to test the robustness of the results.

To compare the performance of penKid to that of daily urinary output ($>$ 436 ml/d vs. $\leq$ 436 ml/d) and to investigate whether the randomization group (Systemic Heparin Anticoagulation vs Regional Citrate Anticoagulation) might also affect the competing outcomes, we estimated CIFs and fitted univariate Fine & Gray models in the same manner as for penKid. In search of a suitable cut-off for daily urinary output, we repeated the sensitivity analysis as described above. To examine whether our results were robust to changes in the relapse-free period chosen in the outcome definition, we repeated the main analyses with a relapse-free period of 2 and 90 days.

Finally, we fitted a multivariable Fine & Gray model with penKid, age and diabetes predicting "successful liberation from CRRT" at landmark analysis to adjust our main result on potentially confounding baseline characteristics. These were selected by statistically significant differences between penKid groups at landmark.
